# Metformin reverse minocycline to inhibit minocycline-resistant *Acinetobacter baumannii* by destroy the outer membrane and enhance membrane potential *in vitro*

**DOI:** 10.1186/s12866-022-02629-4

**Published:** 2022-09-12

**Authors:** Tingting Guo, Xiaoli Sun, Jie Yang, Liying Yang, Mengying Li, Yuhang Wang, Hongmei Jiao, Guocai Li

**Affiliations:** 1 Department of Microbiology, Medical College, Yangzhou University, Yangzhou, 225001 China; 2 Jiangsu Key Laboratory of Zoonosis/Jiangsu Co-Innovation Center for Prevention and Control of Important Animal Infectious Diseases and Zoonoses, Yangzhou University, Yangzhou, 225009 China; 3 Jiangsu Key Laboratory of Integrated Traditional Chinese and Western Medicine for Prevention and Treatment of Senile Diseases, Yangzhou, 225001 China; 4 Department of Pharmacy, Suzhou Hospital of Integrated Traditional Chinese and Western Medicine, Suzhou, 215101 Jiangsu China

**Keywords:** *Acinetobacter baumannii*, Metformin, Minocycline, Potentiator, Mechanisms

## Abstract

**Background:**

*Acinetobacter baumannii (A. baumannii)* is an opportunistic pathogen and has emerged as one of the most troublesome pathogens. Drug resistance in *A. baumannii* has been reported on a global scale. Minocycline was found to be active against multi-drug resistant *A. baumannii* and was approved by the FDA for the infections caused by sensitive strains of *A. baumannii.* However, the emergence of minocycline resistance and its toxic effects still need to be addressed. Therefore, this study aimed to evaluate the synergistic effects of metformin combined with minocycline on minocycline-resistant *A. baumannii*.

**Results:**

The effect of metformin on the antibacterial activity of minocycline was determined by checkerboard and time-killing assay. Further, it was observed by biofilm formation assay that metformin combination with minocycline can inhibit the formation of biofilm. Outer membrane integrity, membrane permeability, membrane potential and reactive oxygen species (ROS) were monitored to explore the underlying synergistic mechanisms of metformin on minocycline. And the results shown that metformin can destroy the outer membrane of *A. baumannii*, enhance its membrane potential, but does not affect the membrane permeability and ROS.

**Conclusion:**

These findings suggested that the combination of metformin and minocycline has the potential for rejuvenating the activity of minocycline against minocycline-resistant *A. baumannii*.

## Background

Bacterial infections have been a serious global threat owing to the increasing prevalence of multidrug-resistant (MDR) Gram-negative bacteria [1]. Although *A. baumannii* is an opportunistic nosocomial pathogen causing ventilator-associated pneumonia (VAP), blood infections, and urinary tract infections [2], it is now resistant to antimicrobials agents, including carbapenems, leading to the evolution of multidrug-resistant (MDR) strains like Carbapenem-resistant *A. baumannii* (CR-Ab) that has become a major public health concern [3]. Minocycline as an old antibiotic is relatively safe and low cost [4]. Because of limited antibiotic choices and difficultics to develop novel antimicrobial agents, minocycline has been used for the treatment of drug-resistant *A. baumannii* [4, 5] and is often used in combination with other antibiotics [4, 6]. As minocycline is a second-generation semisynthetic tetracycline analog [7], its primary mechanism involves attachment to the bacterial 30S ribosomal subunit, thereby affecting the protein synthesis and inhibiting the bacterial peptide formation and further bacterial growth [8]. Minocycline is only a substrate of tetB and can escape other tetracycline efflux pumps [9, 10]. Minocycline could result in significant derangements in the microbiota of the skin and gut and potentially lasting the effects on bone metabolism [11, 12]. Since approximately 20% of *A. baumannii* isolates are resistant to minocycline [13], antibiotic adjuvant that may reduce the dosage of antibiotics and reduce toxicity could be a promising approach [14]. Combining antibiotics with non-antibiotic adjuvants has been seen to be successful in overcoming resistance against β-lactam resistant Gram-negative pathogens [15]. Besides this, cationic antimicrobial polymers can also enhance the antibacterial activity of polypeptide antibiotics [16].

**Fig. 1:**
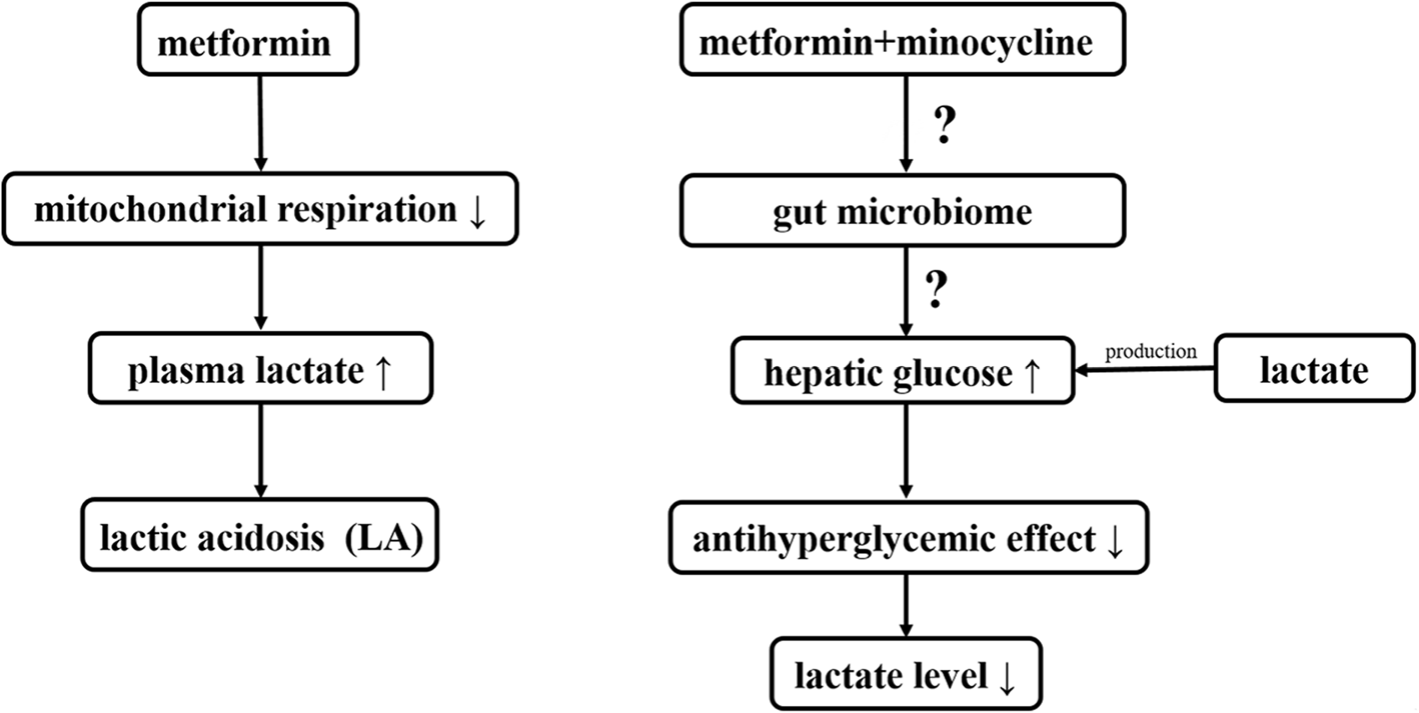
Diagrammatic representation depicting hypothetical view of the metformin and minocycline combination in culminating lactic acidosis. Metformin can inhibit liver mitochondrial respiration, thus increasing plasma lactate levels. Metformin-associated lactic acidosis (MALA) can be caused by elevated plasma metformin concentrations and disrupted lactate production or clearance. A decrease antihyperglycemic effect of metformin maybe observed after concomitant administration with minocycline because of gut microbiome change, and may cause a decrease production of lactate. This hypothetical need more research to prove

Metformin is the most popular orally administered drug used for lowering blood glucose worldwide that is widely considered to be the preferred initial treatment of patients with type 2 diabetes mellitus [17]. Metformin can inhibit hepatic gluconeogenesis, thus exerts its function of glucose-lowering. In addition, the inhibition of mitochondrial complex I by metformin lead to cAMP and protein kinase A signalling defectively, thus opposing the action of glucagon [18]. Metformin is generally considered as a safe and well-tolerated medication [19]. The main adverse effects of metformin being reported is lactic acidosis, and the mechanism of this is because of the inhibition of hepatic glucose production from lactate molecules lead to the accumulation of lactate [20]. According to the research of *Kim et.al*, a decrease antihyperglycemic effect of metformin was observed after concomitant administration with vancomycin because of gut microbiome change [21]. The decrease antihyperglycemic effect caused by combination of metformin with vancomycin may also causing a decrease production of lactate. The combination of metformin with minocycline may have a similar phenomenon. This need more research to study whether this combination could bypass this common side effects. The diagrammatic representation depicting hypothetical view of the metformin and minocycline combination in culminating lactic acidosis is shown in Fig. 1. Previous research has shown that metformin has several innate properties like anti-cancer, immunoregulatory, and anti-aging effects, which is now attracting the attention of researchers in varied fields other than endocrinology [22]. It was reported that diabetic patients treated with metformin were less likely to have bacterial infections, revealing a potential antimicrobial function of metformin [23, 24] which was also substantiated by the findings of Singhal A *et al*. that metformin was capable of reducing the intracellular growth of *Mycobacterium tuberculosis* depending on the AMPK (adenosine monophosphate-activated protein kinase) energy sensor [25]. Liu Y *et al.* stated that metformin effectively potentiates doxycycline against tet(A)-positive *E. coli* B2 strains and could moderately activate innate immune response [26]. Whereas *in vitro* results of another study depicted that metformin efficiently down-regulated the quorum sensing of *Pseudomonas aeruginosa*, thus, inhibiting the bacterial motility and biofilm formation [27].

In this study, as we explored the synergistic activity of metformin in combination with antibiotics against *A. baumannii*, our findings indicated that metformin synergistically in combination with minocycline inhibits biofilm formation, destroys the outer membrane integrity, and increases the membrane potential.

## Results

### Synergistic activity of metformin with antibiotics

**Fig. 2:**
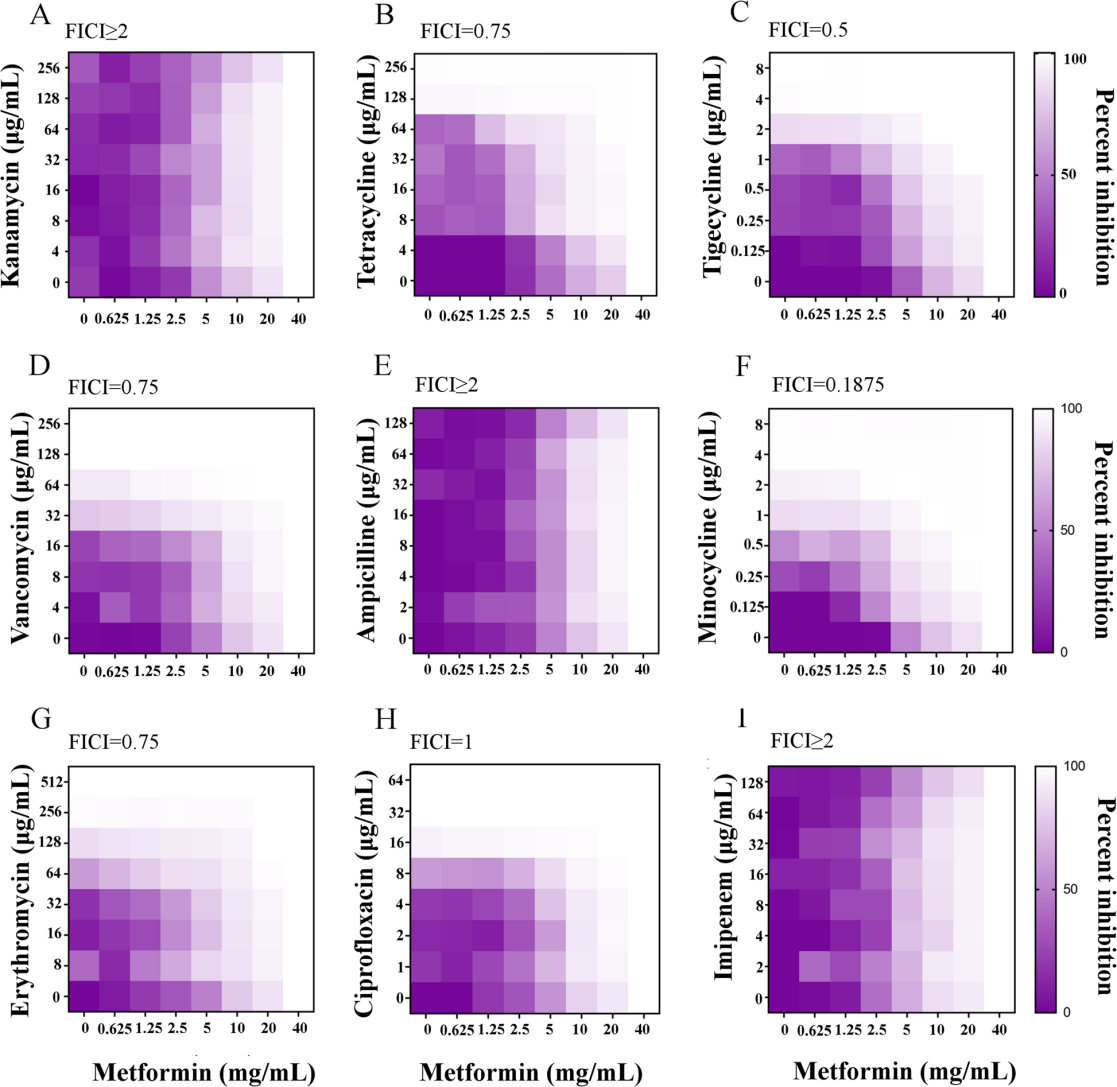
Checkerboard assays of metformin with different classes of antibiotics against *A. Baumannii* A1 strain. The primary screening indicated that metformin had a synergistic activity with minocycline against *A. Baumannii* A1 strain. **A** Metformin in combination with kanamycin, FICI ≥ 2; **B** Metformin in combination with tetracycline, FICI = 0.75; **C** Metformin in combination with tigecycline, FICI = 0.5; **D** Metformin in combination with vancomycin FICI = 0.75; **E** Metformin in combination with ampicillin, FICI ≥ 2; **F** Metformin in combination with minocycline, FICI = 0.1875; **G** Metformin in combination with erythromycin, FICI = 0.75; **H** Metformin in combination with ciprofloxacin, FICI = 1; **I** Metformin in combination with imipenem, FICI ≥ 2. The synergy is defined as FICI ≤ 0.5; irrelevance is defined as 0.5 < FICI ≤ 4; and antagonism is defined as FICI > 4

In order to determine the efficacy of metformin in combination with antibiotics inhibits multidrug-resistant *A. baumannii,* the synergistic antimicrobial activity of metformin combined with several antibiotics was investigated by chequerboard broth microdilution assays. The aim of chequerboard broth microdilution assays using different classes of antibiotics against *A. baumannii* A1 was to test whether the synergy is minocycline-specific. The activities of nine antibiotics (kanamycin; tetracycline; tigecycline; vancomycin; ampicillin; minocycline; erythromycin; ciprofloxacin; imipenem) with metformin against *A. baumannii* isolate A1 is shown in Fig. 2. Metformin showed a synergistic effect with minocycline against *A. baumannii* A1 through primary screening, the results displayed that the metformin was a potential adjuvant to minocycline with Fractional Inhibitory Concentration Index (FICI) = 0.1825, Fig. 2 F). The metformin activity in conjunction with different antibiotic classes against *A. baumannii* A1 was enumerated as follows; the FICI (kanamycin with metformin) ≥ 2; FICI (tetracycline with metformin) = 0.75; FICI (tigecycline with metformin) = 0.5; FICI (vancomycin with metformin) = 0.75; FICI (ampicillin with metformin) ≥ 2; FICI (minocycline with metformin) = 0.1875; FICI (erythromycin with metformin) = 0.75; FICI (ciprofloxacin with metformin) = 1; FICI (imipenem with metformin) ≥ 2, thereby implying that FIC indices of metformin with tigecycline, tetracycline, ampicillin, kanamycin, imipenem, vancomycin, erythromycin, and ciprofloxacin were above > 0.5, suggesting irrelevance or antagonism as shown in Fig. 2.

**Fig. 3:**
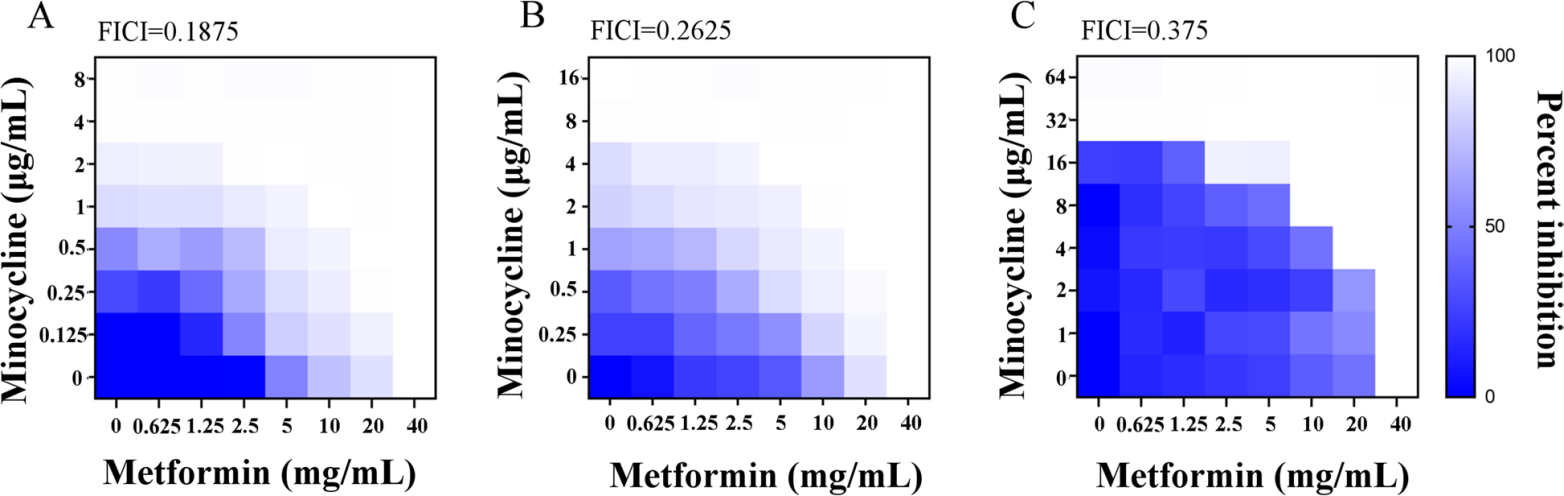
Synergistic activity of metformin with minocycline against different strains of *A. baumannii*. **A** Metformin in combination with minocycline against *A. baumannii* A1 strain, FICI = 0.28125; **B **Metformin in combination with minocycline against *A. baumannii* A163 strain, FICI = 0.25; **C **Metformin in combination with minocycline against *A. baumannii* A156 strain, FICI = 0.375

As *A. baumannii* A1 strain was sensitive to minocycline (Table 1), the evaluation of metformin’s synergistic effect with minocycline on A163 (minocycline-mediated strain) and A156 (minocycline-resistant strain) depicted that metformin also had a synergistic effect on *A. baumannii* A163 and A156 strains (Fig. 3). Since metformin FICI with minocycline for A163 and A156 was 0.375 and 0.1825, respectively, it indicated that the synergistic effect of metformin with minocycline existed in *A. baumannii* strains no matter it is minocycline resistant or not.

**Table 1:** Antimicrobial susceptibility test of strains used in this study (MIC, μg/mL). R represent resistance. I represent intermediate. S represent sensitive

Antibiotics(μg/ml)	Strains		
	A1	A163	A156
Doxycycline	32(R)	64(R)	64(R)
**Minocycline**	**4(S)**	**8(I)**	**32(R)**
Tigecycline	4(S)	0.5(S)	16(R)
Ceftriaxone	≥ 512(R)	≥ 512(R)	≥ 512(R)
Imipenem	≥ 1024(R)	≥ 1024(R)	≥ 1024(R)
Gentamicin	≥ 1024(R)	≥ 1024(R)	≥ 1024(R)
Kanamycin	≥ 1024(R)	≥ 1024(R)	≥ 1024(R)
Ciprofloxacin	32(R)	64(R)	≥ 128(R)
Polymyxin B	2(S)	2(S)	4(S)
Colistin	0.25(S)	0.125(S)	0.25(S)
Erythromycin	512(R)	512(R)	128(R)
Rifampicin	1(S)	2(S)	32(R)
Tetracycline	256(R)	128(R)	256(R)
Ampicillin	≥ 1024(R)	≥ 1024(R)	≥ 1024(R)
Vancomycin	128(R)	64(R)	≥ 1024(R)

**Fig. 4:**
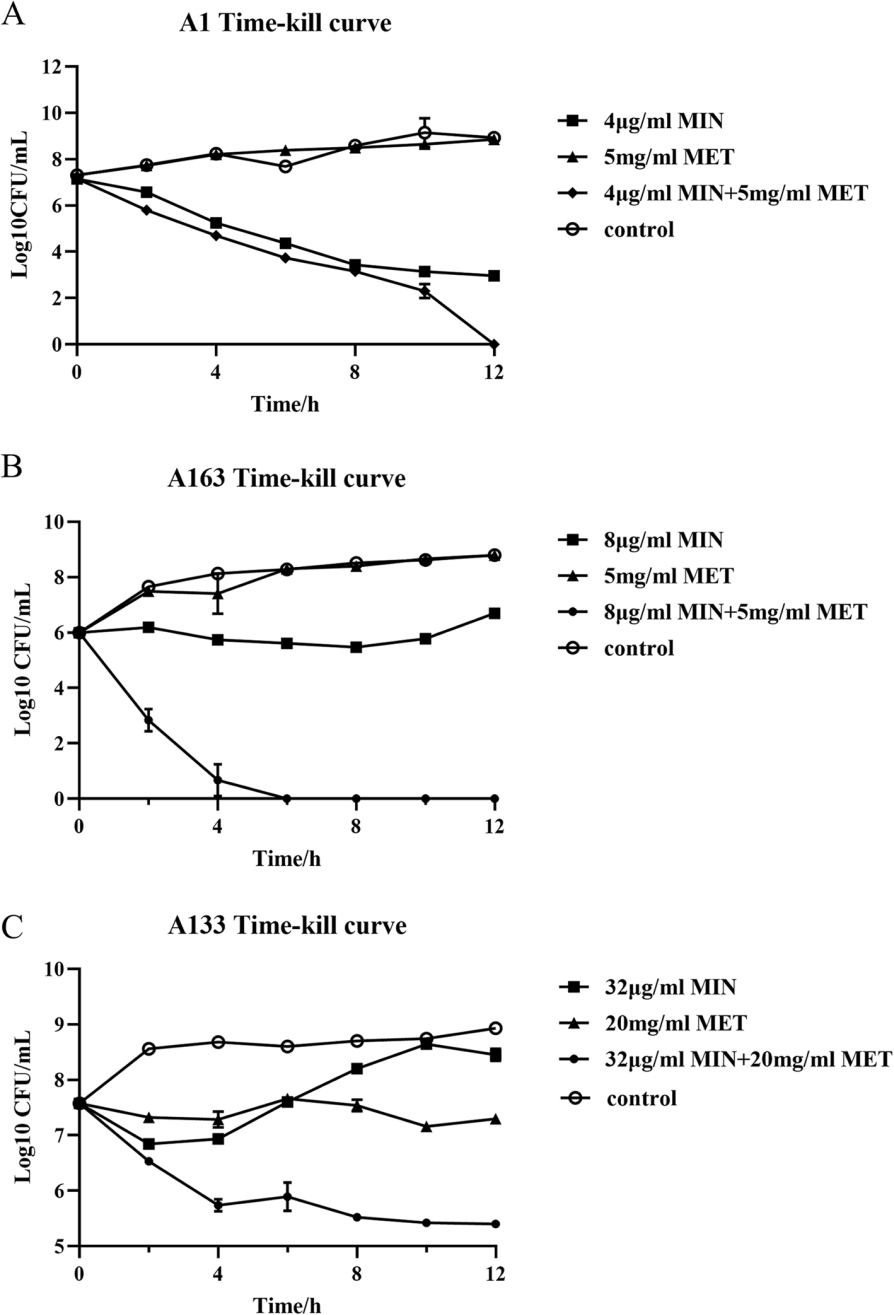
Time-dependent killing of *A. baumannii* by the combination of minocycline and metformin. MIC of minocycline and 0.25 × MIC of metformin of strains were selected as treatment concentrations. **A** minocycline (MIN, 4 µg/mL) or metformin (MET, 5 mg/mL) alone or in combination (MIN + MET, 4 µg m/L + 5 mg/mL). **B** minocycline (MIN, 8 µg/mL) or metformin (MET, 5 mg/mL) alone or in combination (MIN + MET, 8 µg m/L + 5 mg/mL). **C** minocycline (MIN, 32 µg/mL) or metformin (MET, 10 mg/mL) alone or in combination (MIN + MET, 32 µg/mL + 10 mg/mL). Data are representative of three biological replicates and presented as mean ± SD

Time-dependent killing curves were determined using strains with different sensitivity to minocycline for studying the synergistic bactericidal activity of metformin with minocycline. And the results showed that combination of metformin with minocycline displayed obvious bactericidal activities against *A. baumannii* (Fig. 4).

### Metformin inhibited the formation of biofilms

**Fig. 5:**
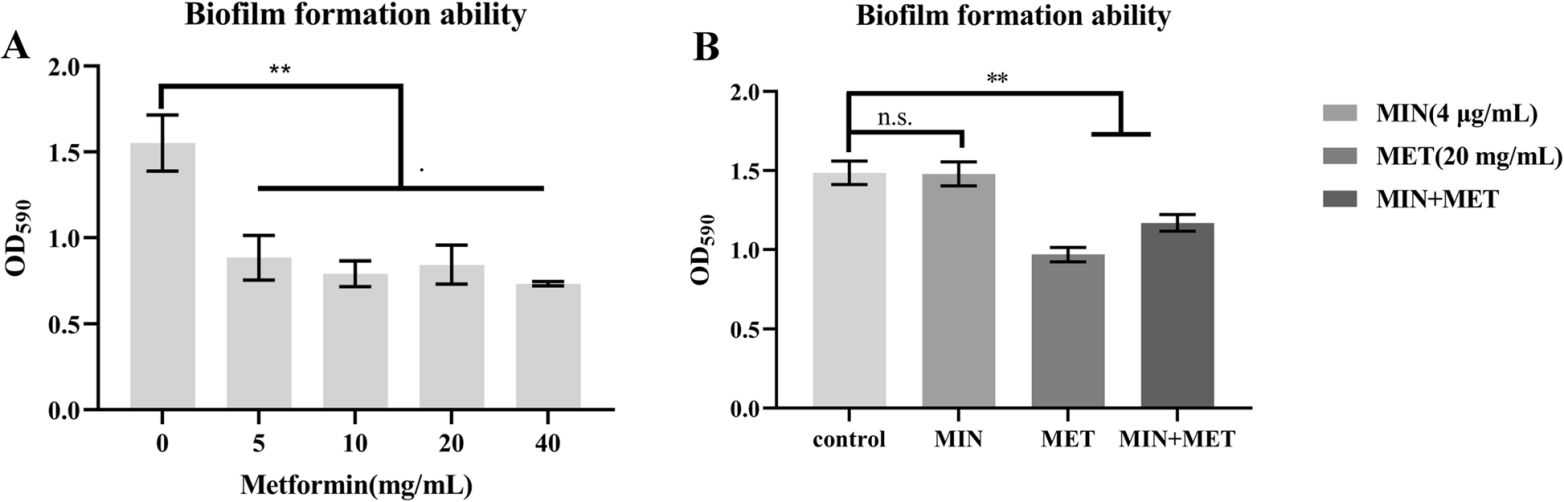
Metformin can inhibit biofilm formation and minocycline does not affect the biofilm formation of *A. baumannii* A156. **A** The effect of different concentrations of metformin on the ability of biofilm formation; **B** The effect of metformin, minocycline, or minocycline plus metformin on the biofilm formation. Data are presented as mean ± SD, (*p ≤ 0.05, **p < 0.01)

After confirming that metformin potentiates minocycline killing against *A. baumannii*, we sought to conduct the potential mechanism studies on minocycline resistant *A. Baumannii* A156 strain. Biofilm can protect microorganisms and enhance their resistance against stressed environments and antibiotics [28, 29]. Biofilm formation assay was performed to determine the efficacy of metformin combination with minocycline for inhibiting the biofilm formation, confirming that metformin can inhibit the formation of biofilms. However, minocycline itself has no effect on biofilm formation (Fig. 5 A, B).

### Metformin destroyed outer membrane of *A. baumannii*

**Fig. 6:**
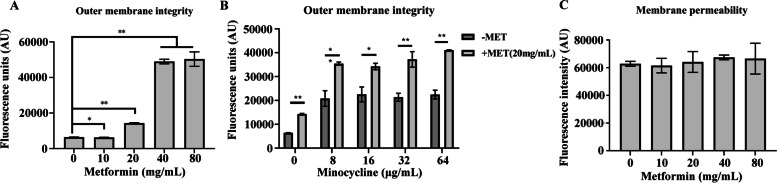
The effect of metformin on the outer membrane permeability and membrane integrity of *A. baumannii* A156. **A** Metformin destroys outer membrane permeability with the increasing concentrations of metformin used. **B** No effect on outer membrane permeability in *A. baumannii* with the treatment of minocycline alone; however, when metformin was added, the outer membrane permeability significantly increased. **C** No effect on membrane integrity after treatment with metformin. Data are presented as mean ± SD, *p ≤ 0.05; **p ≤ 0.01

Both NPN and PI were used to detect the outer membrane permeability and membrane integrity for studying whether the intracellular minocycline accumulation induced by metformin is due to the change of membrane permeability in *A. baumannii*; the results are shown in Fig. 6. It is reported that metformin increased outer membrane permeability in a concentration-dependent manner (Fig. 6A) while the addition of different minocycline concentrations did not influence the outer membrane permeability of *A. baumannii*; however, the outer membrane permeability of *A. baumannii* increased significantly when metformin was added (Fig. 6B). However, different concentrations of metformin does not affect the membrane permeability of *A. baumannii* (Fig. 6C)*.* Hence, these results indicated that metformin in conjunction with minocycline might only lead to outer membrane damage.

### Metformin can enhance the membrane depolarization of *A. baumannii*

**Fig. 7:**
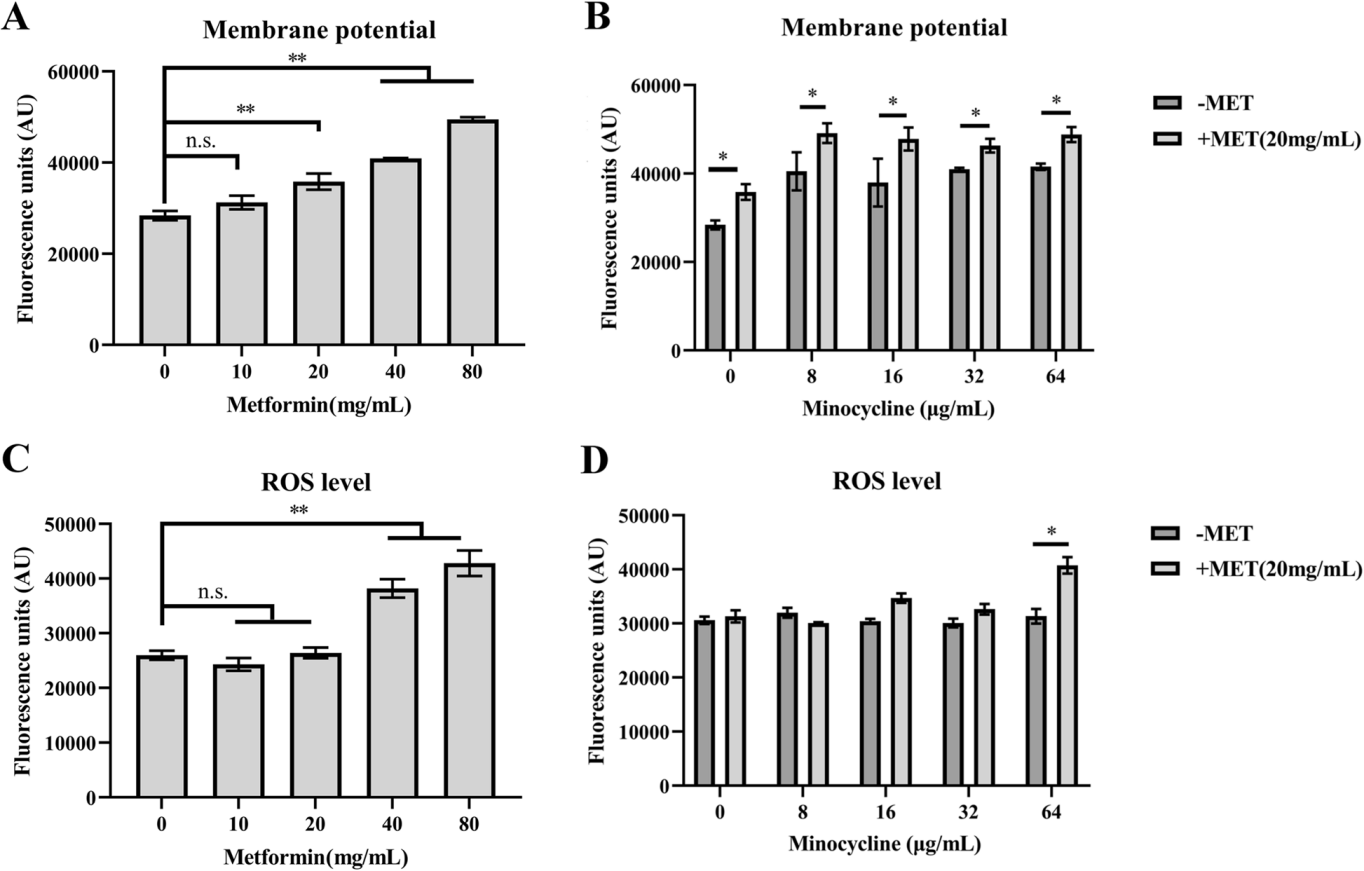
The effect of metformin on the membrane potential and ROS production of *A. baumannii* A156*.* **A**, **B** Metformin causes dissipation of membrane potential and minocycline does not affect membrane potential. **C**, **D** Increased generation of ROS were detected only in high concentrations of metformin. Minocycline alone or plus metformin do not affect ROS level. Data are expressed as the mean ± SD of three replicates, *p ≤ 0.05; **p ≤ 0.01

The damaged outer-membrane induced by metformin might cause dysfunctions in cytoplasmic membrane; thus, DiSC_3_(5) was used to detect the bacterial membrane depolarization. The *A. baumannii* treatment with 20 mg/mL,40 mg/mL and 80 mg/mL metformin showed an increase (*p* < 0.05) in fluorescence, whereas using 10 mg/mL metformin showed no change (Fig. 7A). The addition of different concentrations of minocycline combined with 20 mg/mL metformin influences the membrane depolarization of *A. baumannii* (Fig. 7B) and further indicated that metformin increased the damage of the outer membrane and dissipated the cytoplasmic membrane potential while no change was observed in whole membrane permeability. Thereby, implying that the structural integrity of the inner membrane might be largely maintained, although the functionality was affected by the usage of these drugs.

### Effect of metformin on the production of ROS

As depicted in previous studies, antibiotics affected many cellular targets by causing a disturbance in metabolism and respiration by generating reactive oxygen species (ROS) [30]. Hence, metformin, minocycline, and a combination of both the drugs were examined for inducing ROS production. The ROS levels of *A. baumannii* A156 were detected and shown in Fig. 7 C and D. It was observed that the minocycline monotherapy group did not display any change of fluorescent intensity when compared with the control and metformin monotherapy groups that showed an increased ROS production only with higher concentrations (40 mg/mL and 80 mg/mL), respectively. While the combination group showed little changes with increasing minocycline concentration combined with 20 mg/mL metformin, only 64 µg/mL minocycline with metformin showed a significant difference (Fig. 7 D); thus, proving that the synergistic effect of metformin and minocycline is not associated with ROS production.

### Resistance development studies

**Fig. 8:**
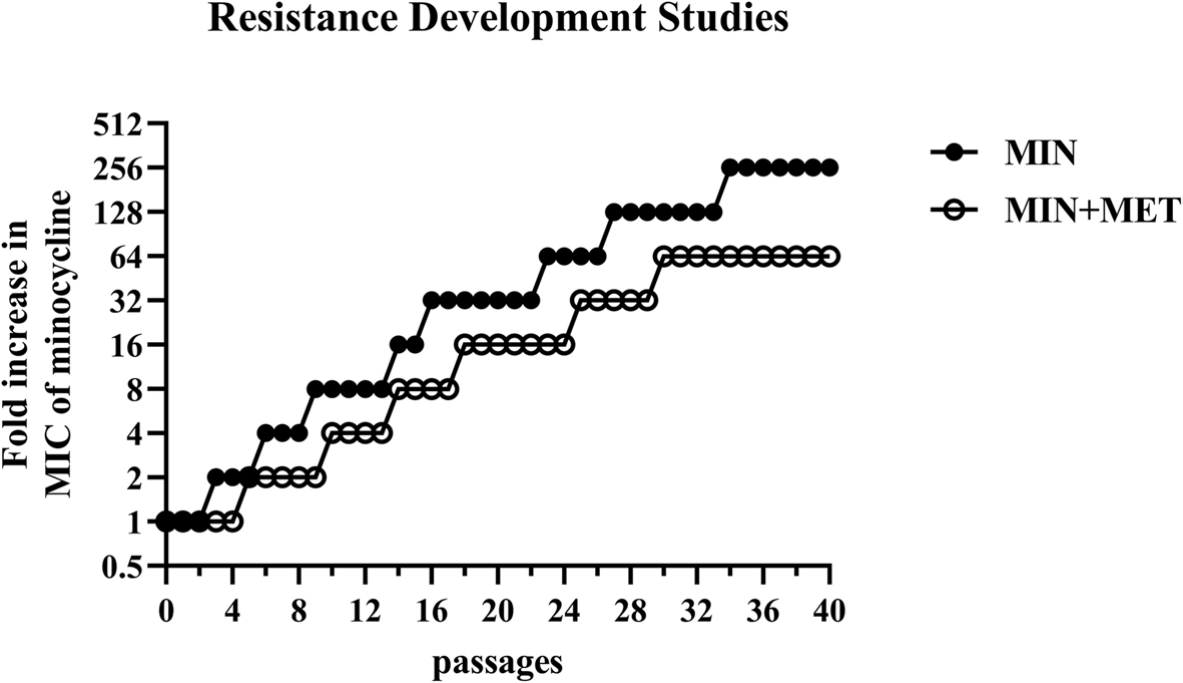
The addition of metformin (10 mg/mL, one-half of MIC) prevents the evolution of minocycline resistance to *A. baumannii* ATCC19606 *in vitro*

In order to further understand the ability of metformin to inhibit the development of antibiotic resistance, resistance development studies were conducted. Due to the multiple targets of metformin action (biofilm formation, membrane damage, and membrane depolarization), the speed of minocycline resistance development against *A. baumannii* might be decreased. 40 passages of ATCC19606 (the standard strain of *A. baumannii*) with minocycline sub-MIC (0.5 × MIC) in metformin’s presence and absence (10 mg/mL, corresponding to half of MIC) was performed. As it was observed that the speed of minocycline resistance development of the minocycline alone group was faster than the combination group (Fig. 8), the metformin’s addition inhibited the evolution of minocycline resistance to *A. baumannii* ATCC 19606 *in vitro* and indicated that the combination of minocycline and metformin could delay the emergence of minocycline resistance.

### Safety evaluation of minocycline and metformin combination

**Fig. 9:**
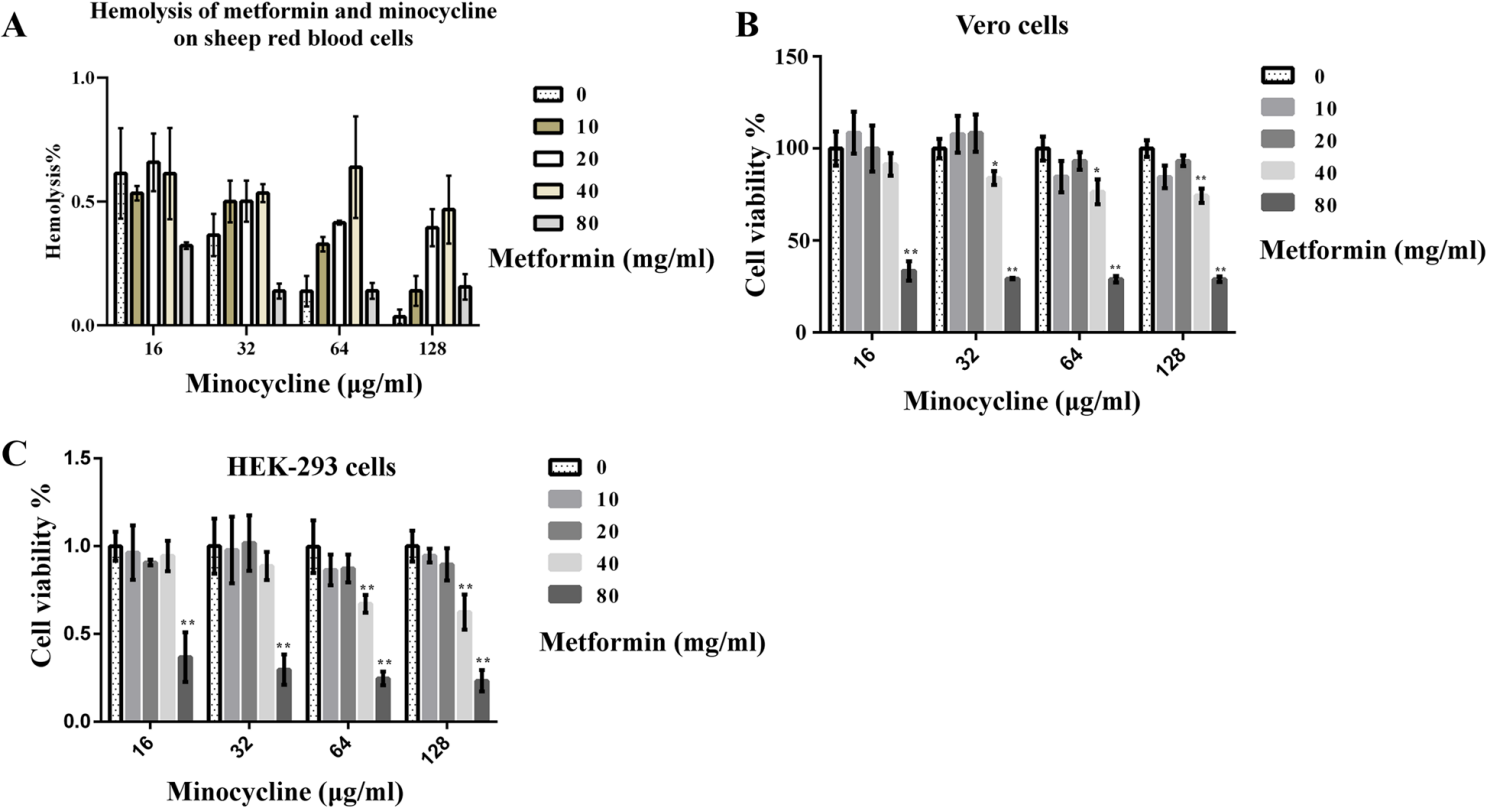
Safety assessment of metformin in combination with minocycline. **A** Hemolytic activity of minocycline to the RBCs in the absence or presence of metformin. Sheep blood cells were treated with combinations of minocycline (16–128 µg/mL) and metformin (0–80 mg/mL). The hemolytic activity of minocycline was less than 5% either alone or in combination with metformin. **B** *In vitro* mammalian toxicity of minocycline in combination with metformin in vero cells using CCK-8 assay. **C** *In vitro* mammalian toxicity of minocycline in combination with metformin in HEK-293 cells using CCK-8 assay. Metformin (≤ 20 mg/mL) combination with different concentration minocycline had no cytotoxicity to vero cells and HEK-293 cells. Cytotoxicity phenomenon were found when using metformin ≥ 40 mg/mL. The data shown here are mean ± SD of three replicates

The hemolysis of minocycline in the presence of metformin was examined. Figure 9A showed that minocycline combination with metformin had negligible hemolytic activity (< 5%), because hemolytic activity below 5% was generally regarded as safe [31]. As renal impairment can lead to the increase of plasma metformin concentration, which may cause metformin-associated lactic acidosis (MALA). Thus, cells of renal origin were used to detect the cell cytotoxicity of metformin in combination with minocycline. The results of CCK-8 assay shown that metformin (≤ 20 mg/mL) combination with different concentration minocycline (16–128 µg/mL) had no cytotoxicity to vero cells and HEK-293 cells (Fig. 9 B and C). However, cytotoxicity phenomenon were found when using metformin ≥ 40 mg/mL,which was different with the results of hemolytic activity. Thus, the use of metformin combination with minocycline is relatively safe when using metformin ≤ 20 mg/mL for further usage in relevant conditions.

## Discussion

The increase of multidrug-resistant (MDR) bacteria has become a severe problem worldwide due to persistent over usage of antibiotics [32]. *A. baumannii*, an opportunistic Gram-negative pathogen, is frequently involved in nosocomial infection outbreaks [33]. In the last two decades, *A. baumannii* infection has been seen to increase in both the incidence and severity in critically ill patients [34]. Thus, it is important to find compounds that increase the effectiveness of antimicrobial agents on drug-resistant bacteria. Several studies have reported that some compounds could enhance the antibacterial activity of antibiotics *via* a number of different mechanisms [35, 36]. Metformin is the first-line treatment for type 2 diabetes mellitus. Although multiple biofunctions of metformin in treatment of diseases such as cardiovascular disease, neurodegenerative disease, cancer, and aging have been demonstrated [37–39], its potential antibacterial activity in bacterial infections has not been fully explored.

Our results revealed that metformin effectively potentiates minocycline against *A*. *baumannii* due to the implementation of several evaluation assays like the checkerboard test assay, whose primary screening identified that metformin had a synergistic activity with minocycline against *A. baumannii* along with the utilization of time-kill kinetics assay that provided significant evidence regarding the synergistic effect of the metformin combination with minocycline, inhibiting the formation of biofilm; thus, reducing the ability of *A. baumannii* to antibiotic resistance. It was also confirmed that metformin in combination with minocycline inhibited *A. baumannii* by destroying the outer membrane, hence enhancing its membrane potential.

Compared to free-swimming (planktonic) bacterial cells, biofilms depicted a higher resistance to killing by a great majority of antimicrobial agents [40] as well as in the realization of biofilm-specific therapy that promoted the dispersal of biofilm cells and might be used later to treat such challenging infections [41]. This study exhibited that metformin inhibited the formation of biofilm, thereby reducing the ability of *A. baumannii* to resist minocycline. Liu Y *et al.* revealed that metformin could destroy gram-negative bacterial outer membrane and dissipated membrane potential of the cytoplasmic membrane [26], which was similar to the inhibitory effect of metformin on the mitochondrial complex –I, the largest multi-subunit complex of the respiratory chain [42]. Metformin might also inhibit bacterial NADH dehydrogenase-like complex type-1 (NDH-1) complex due to a similarity between its structure and function with mitochondrial complex –I [25, 43, 44].

This is the first study that has employed the co-application of metformin and minocycline against *A. baumannii* and provided the evidence of antibacterial potential for using the above-mentioned compounds as antibiotic adjuvants in the treatment of *A. baumannii* as these metformin antibacterial mechanism findings supply a basis for allowing the screening for other antibiotic adjuvants. Nevertheless, more research work on metformin’s synergistic pathway is the need of the hour to provide a better understanding of its potential synergistic mechanism in combating bacterial resistance.

## Conclusion

In conclusion, our research shown that there is a synergistic effects of non-antibiotic drug (metformin) with minocycline on *A. baumannii* that resist minocycline. Mechanism studies proved that metformin can destroy the outer membrane of *A. baumannii*, enhance its membrane potential, but does not affect the membrane permeability and ROS. Therefore, the use of metformin together with minocycline may be a good strategy in clinical treatments and provides a theoretical basis for screening more non-antibiotic drugs in minocycline-based combination.

## Methods

### Bacterial strains and reagents

The employed strains*, A. Baumannii* strains A1 (minocycline-sensitive strain), A163 (minocycline-mediated strain), and A156 (minocycline-resistant strain), were clinically isolated from Zhangjiagang people’s hospital, Xuyi hospital, and Affiliated Hospital of Yangzhou University, respectively and preserved in the pathogenic microbiology laboratory of Yangzhou University while the strains were grown in Mueller–Hinton (MH) broth or on MH agar. Metformin were obtained from Macklin (Shanghai,China), minocycline were obtained form Solarbio (Beijing, China), other antibiotics were obtained from Sangon Biotech (Shanghai, China).

### Susceptibility testing

According to the Clinical & Laboratory Standards Institute (CLSI) 2020 guidelines [45], antibiotic MICs were measured using the standard broth microdilution method in which antibiotics were two-fold diluted in MHB and mixed with the same volume bacterial suspension (≈10^5^ CFU/mL) in a 96-well microtiter plate while the MIC values were defined as the minimum antibiotic concentrations at which bacterial growth is completely inhibited after 18 h incubation at 37 °C.

### Checkerboard assays

The synergistic activities of antibiotics in conjunction with metformin were evaluated by checkerboard assays according to a previously study [45]. The steps are as follows: dispensing of 100 µL MHB into each well of a 96-well plate titer along with the dilution of antibiotics along the ordinate while the metformin was diluted along the abscissa. After 18 h of co-incubation with 100 µL of *A. baumannii* culture (5 × 10^6^ CFUs/well), the optical density (OD) of bacterial culture at 600 nm was measured using a microplate reader. The FIC indices (FICIs) were calculated according to the formula as follows:

$$\mathrm{FIC}\;\mathrm{index}\;=\;{\mathrm{MIC}}_{\mathrm{ab}}/{\mathrm{MIC}}_{\mathrm a}+\;{\mathrm{MIC}}_{\mathrm{ba}}/{\mathrm{MIC}}_{\mathrm b}\;=\;{\mathrm{FIC}}_{\mathrm a}+{\mathrm{FIC}}_{\mathrm b}\;$$

The synergy is defined as FICI ≤ 0.5; irrelevance is defined as 0.5 < FICI ≤ 4; the antagonism is defined as FICI > 4 [46].

### Time-dependent killing curve

Time-kill kinetic assays were carried out with *A. baumannii* strains A1, A163, and A156 for further confirmation of the metformin synergism with minocycline. In brief, overnight *A. baumannii* were diluted 1/1000 in Mueller Hinton Broth (MHB), and then incubation was performed for four hours (exponential phase) at 37 °C. Then the cultures were treated with Phosphate buffered saline (PBS), minocycline (MIN, 4, 8, 32 µg/mL) or metformin (MET, 5, 5, 10 mg/mL) alone or in combination (MIN + MET, 4 µg/mL + 5 mg/mL, 8 µg/mL + 5 mg/mL or 32 µg/mL + 10 mg/mL). The minocycline concentration in this experiment corresponded to the MIC of each strain, while the metformin concentration used was 0.25 MIC of each strain. After the removal of 100 µL bacteria culture aliquots every two hours, succeeded by centrifugation, PBS resuspension, and ten-fold serial dilution, the dilutions were placed on MHA plates. And the colony counts were determined after incubation for 12 h at 37 °C. Performed experiments were conducted in triplicate, and the mean ± SD was revealed accordingly.

### Biofilm inhibition assay

The biofilm formation assay was conducted in a 96-well plate as previously described [47]. First, *A. baumannii* A156 isolates were cultured in MH (5 mL) at 37 °C with a shaking rate of 200 rpm for 12 h. Then, adding 100 µL of a mixture containing different concentration of only metformin (metformin, 0–40 mg/mL), only minocycline (4 µg/mL) as well as a combination of both drugs (metformin 20 mg/mL; minocycline 4 µg/mL) to each well. Furthermore, as 100 µL of the prepared bacterial culture containing 1 × 10^6^ CFU/mL was added to each well and incubated at 37 °C for 24 h. At last, planktonic cells were removed, and the wells were washed with PBS and air-dried followed by the staining of the adhered biofilm cells with 1% crystal violet for 30 min and repeated PBS rinse thrice. Thenceforth, as the wells were dissolved in ethanol (95%), the experiment was repeated thrice, followed by measurement of absorbance at 590 nm using Bio-Tek SYNERGY2 (Bio-Tek, USA).

### Outer membrane integrity and membrane permeability

In order to confirm the outer membrane integrity and membrane permeability due to the combination of metformin with minocycline, the bacterial was investigated by the usage of 1-N-phenylnaphthylamine (NPN) and propidium iodide (PI) [45, 48]. First, the bacteria were collected, washed with PBS and suspended to 1 × 10^7^ CFU/mL in PBS followed by treatment with metformin (final concentrations 0–80 mg/mL) for testing the singular effect of the metformin on the outer membrane. Furthermore, in another group, the bacterial suspensions were treated with minocycline (MIN,0–64 µg/mL) or metformin (MET, 20 mg/mL) alone or in a combination for one hour at 37 °C 180 rpm using untreated bacteria as the control. For outer membrane integrity detection, the fluorescence intensity of the bacterial suspension (200 µL) as well as 10 µM NPN (Aladdin,Shanghai,China) in 96-well black microtiter plate was immediately measured at the excitation and emission wavelengths of 350 and 420 nm, respectively. For membrane permeability detection, the measurement of fluorescence intensity of 10 nM PI (Thermo Fisher Scientific)-labeled bacterial cells treated with drugs using the excitation and emission wavelength of 535 and 615 nm, respectively.

### Membrane depolarization

Membrane depolarization of *A. baumannii* were determined using 3, 3-dipropylthiadicarbocyanineiodide (DiSC_3_(5)) according to a previously study [49]. Bacteria were collected, washed, and suspended in 5 mM HEPES [pH 7.0, with 5 mM glucose] with an optical density (OD600) of 0.5. Then, pre-treatment bacteria with metformin (0–80 mg/mL) and minocycline (0–64 µg/mL) alone or combined with 20 mg/mL metformin for one hour at 37 °C while using untreated bacteria as the control. As the membrane potential was measured by 3, 3-dipropylthiadicarbocyanine iodide (DiSC_3_(5), 0.5 µM) (Yuanye, Shanghai, China), the membrane potential level of bacterial cells was measured with the excitation and emission wavelength of 622 and 670 nm, respectively.

### ROS level

In order to measure the generation of reactive oxygen species (ROS), a fluorescent probe, DCFH-DA (2′,7′-dichlorodihydrofluorescein diacetate; Sigma-Aldrich, USA), was used based on the study of Chen *et al. * [50]. Bacteria were collected, washed with PBS and suspended to 1 × 10^7^ CFU/mL in PBS followed by the treatment of bacterial cells with different concentrations of metformin (0–80 mg/mL) or minocycline (0–64 µg/mL) or its combination with 20 mg/mL metformin followed by addition of 5 µM of DCFH-DA to the bacterial suspension and incubation at 37 °C for 30 min in the dark. At last, the fluorescence intensity was measured with a fluorescence microplate reader using excitation and fluorescence detection at 488 and 530 nm wavelengths, respectively.

### Resistance development studies

Due to the sensitivity of *A. baumannii* isolate, ATCC19606 to minocycline, it was employed further in the resistance development studies. ATCC 19606, after culture at 37 °C for 24 h was diluted in 1:100 MHB media supplement with 0.5 × MIC of minocycline or minocycline plus 0.5 × MIC of metformin (10 mg/mL) which was then cultured again at 37 °C for 24 h. Then, the cultures were diluted in 1:100 MHB, and cultured to an OD600 of 0.5 while determining the MIC by two-fold serial dilutions in 96-well microtiter plates. Furthermore, the cultures were again diluted in 1:100 MHB media containing 0.5 × MIC of drugs for a total of 40 passages containing ATCC19606 inducing minocycline-resistant strains along with the calculation of the fold increase in subsequent minocycline MIC relative to initial MIC [51].

### Safety assessment

The hemolytic activity and mammalian cell cytotoxicity of metformin in combination with minocycline was evaluated based on previously reported studies [46, 52]. For hemolytic activity assay, 8% sheep blood cells (Yuanye, Shanghai, China) prepared from fresh sterile defibrinated sheep blood was treated with a combination of minocycline (16–128 µg/mL) and metformin (0–80 mg/mL), using 0.2% Triton X-100 as a positive control and PBS (0.01 mol/L, pH = 7.4) as a negative control at 37 °C for one hour followed by the measurement of the released hemoglobin absorption at 576 nm by Bio-Tek Synergy2 Multi-mode Microplate Reader. The percentage of hemolysis percentage was calculated by:

$$\mathrm{Hemolysis}\;(\%)\;=\;\lbrack({\mathrm{OD}576}_{\;\mathrm{sample}}-{\mathrm{OD}576}_{\;\mathrm{blank}})/({\mathrm{OD}576}_{\;0.2\%\mathrm{Triton}\;\mathrm X-100}-{{\mathrm{OD}57}6}_{\mathrm{blank}}\;)\rbrack\;\times100\%.$$

To test the mammalian cell cytotoxicity of metformin in combination with minocycline, CCK-8 assay was done using African green monkey kidney (vero) cells and human embryonic kidney (HEK-293) cells. The cytotoxicity assay was done according to the manufacturer’s instructions of the CCK-8 kit (Beyotime, Shanghai, China). First, cells were seeded in a 96-well plate at about 2 × 10^3^ cells/well and cultured for 16–24 h. Then, cells were treated with a combination of different concentration minocycline (16–128 µg/mL) and metformin (0–80 mg/mL) for 24 h with three replicates for each concentration. After 24 h of culture, 10 μl of CCK-8 solution was added to per well and the plate was further incubated for 2 h at 37 °C. Then we checked the absorbance at 450 nm to determine the toxicity of minocycline in combination with metformin to the tested cells.

## Data Availability

The datasets used and/or analyzed during the current study available from the corresponding author on reasonable request.

## Abbreviations

*A. baumannii*: *Acinetobacter baumannii*
MET: Metformin
MIN: Minocycline
ROS: Reactive oxygen species
MH: Mueller–Hinton
NPN: 1-N-phenylnaphthylamine
PI: Propidium iodide
